# Molecular evolution of umami/sweet taste receptor genes in reptiles

**DOI:** 10.7717/peerj.5570

**Published:** 2018-08-24

**Authors:** Ping Feng, Shichu Liang

**Affiliations:** 1 Key Laboratory of Ecology of Rare and Endangered Species and Environmental Protection (Guangxi Normal University), Ministry of Education, Guilin, China; 2 Guangxi Key Laboratory of Rare and Endangered Animal Ecology, Guangxi Normal University, Guilin, China; 3 College of Life Sciences, Guangxi Normal University, Guilin, China

**Keywords:** Taste receptor gene, Umami/sweet, Snakes, Reptiles, Diet, Evolution

## Abstract

Sensory systems play an important role in animal survival. Changes to these systems may be critical in evolution of species in new environments. Previous studies exploring the correlation between feeding ecology and *Tas1r* evolution mainly focused on mammals and birds, and found that the relationship was complex. However, in reptiles, the correlation between *Tas1r* evolution and dietary preferences is still unclear. Here, we attempted to explore this relationship in representative species of the major groups of reptiles (turtles, snakes, lizards, crocodilians), for which the genome information is known. We first predicted the functionality (intact, partial, or defective) of *Tas1r*, and then related it to the feeding preferences. As a result, we identified 11 *Tas1r1*, 12 *Tas1r2*, and 12 *Tas1r3* genes to be partial or intact and another 22 *Tas1r* genes to be absent or pseudogenized in the 19 reptiles. We found that, as it was revealed in some other vertebrate groups, no correlation existed between feeding ecology and *Tas1r* evolution in reptiles: genomic prediction indicated that the *Tas1r* genes possibly have been lost or pseudogenized in snakes, but in crocodylia and testudines *Tas1r* genes are either intact or partial, regardless of their feeding habits. Thus, we suggest that the driving force of *Tas1r* evolution in reptiles is complex, and the feeding habit of swallowing food whole without chewing or the absence of taste buds in certain species may account for the possible umami/sweet perception loss. In addition, we propose that caution should be taken when predicting gene functionality from the publicly available genome database.

## Introduction

Taste perception plays an important role in the survival of animals and their daily life. There are five modalities of taste perception: umami, sweet, bitter, salty, and sour. Umami, sweet, and bitter perception are mediated by G protein-coupled taste receptors with seven transmembrane α-helical regions, and the taste receptors of umami and sweet are encoded by the *Tas1r* family, which is composed of three members (*Tas1r1*, *Tas1r2*, *Tas1r3*) (Nelson et al., 2001), while bitter receptors are encoded by the *Tas2r* family (Nei, Niimura & Nozawa, 2008). Functional assays have demonstrated that Tas1r1 combines with Tas1r3 to form the umami taste receptor, while Tas1r2 + Tas1r3 responds to sweet tastants and functions as sweet taste receptor (Nelson et al., 2001, 2002). *Tas1r* genes include several exons, and the corresponding proteins are distinguished by a long N-terminal domain which may participate in ligand binding (Pin, Galvez & Prezeau, 2003). Taste perception is believed to be closely related to the diet of a species (Li et al., 2005; Shi & Zhang, 2006; Bachmanov & Beauchamp, 2007; Feng & Zhao, 2013); transgenic rescue experiments and behavioral studies have demonstrated that defective taste receptor genes can lead to taste dysfunction (Zhao et al., 2003), which indicates that absence or defect of the receptor genes will result in a disability of taste.

*Tas1r* expansions have been discovered in certain species of fish (Ishimaru et al., 2005), and taste receptor gene losses are also found in other vertebrate species, in some cases the loss is linked with feeding habits. For example, the giant panda, feeding primarily on bamboo, has a pseudogenized *Tas1r1* gene (Li et al., 2010), while cat (*Felis catus*), which is a carnivore, also has a pseudogenized *Tas1r2* gene and exhibits indifference to carbohydrates (Li et al., 2005). Nevertheless, in some cases, the evolution of *Tas1r* does not show strict concordance with feeding ecology. For instance, the horse and cow are herbivorous, but they still have intact *Tas1r1* (Zhao et al., 2010). Besides, it is demonstrated that most birds, such as hummingbird, ground tit, turkey, chicken, penguin, and zebra finch, lack *Tas1r2* (Feng & Zhao, 2013; Baldwin et al., 2014; Zhao, Li & Zhang, 2015), yet hummingbird can taste sweet tastants, suggesting that the correlation between *Tas1r* functionality and feeding ecology is complex in birds.

So far, most research on *Tas1r* has principally concentrated on mammals and birds, because of the higher availability of mammalian and avian genome drafts (Shi & Zhang, 2006; Feng et al., 2014; Jarvis et al., 2014; Liu et al., 2014; Zhang et al., 2014). As multiple genomes from representative of reptiles have been released recently (Castoe et al., 2013; Wan et al., 2013; Wang et al., 2013; Green et al., 2014), species of this group have attracted increasing attention (Khan et al., 2015; Vandewege et al., 2016). However, any potential correlation between feeding ecology and the umami/sweet taste receptor gene evolution in reptiles is still unclear. To fill this gap, we used the recently released genomes of 19 reptiles, including two lizards, eight snakes, four crocodiles, and five turtles to survey *Tas1r* evolution. The study focuses on the following questions: (1) What is the functionality of reptile *Tas1r*; (2) does functionality of *Tas1r* vary among the different lineages of reptiles; and (3) is there a correlation between *Tas1r* functionality and feeding habits in reptiles?

## Materials and Methods

### Data resources

A total of 19 reptile genomes representing two lizards, eight snakes, four crocodiles, and five turtles were downloaded from the National Center for Biotechnology Information database (https://www.ncbi.nlm.nih.gov/). They are Japanese gecko (*Gekko japonicus*), green anole (*Anolis carolinensis*), Burmese python (*Python bivittatus*), king cobra (*Ophiophagus hannah*), corn snake (*Pantherophis guttatus*), common garter snake (*Thamnophis sirtalis*), adder (*Vipera berus*), brown spotted pit viper (*Protobothrops mucrosquamatus*), timber rattlesnake (*Crotalus horridus*), speckled rattlesnake (*Crotalus mitchellii*), saltwater crocodile (*Crocodylus porosus*), gharial (*Gavialis gangeticus*), Chinese alligator (*Alligator sinensis*), American alligator (*Alligator mississippiensis*), spiny softshell turtle (*Apalone spinifera*), Chinese softshell turtle (*Pelodiscus sinensis*), green sea turtle (*Chelonia mydas*), painted turtle (*Chrysemys picta*), and diamondback terrapin (*Malaclemys terrapin*). The sequencing depths and statistics of genomic contig N50 were summarized in Zhong et al. (2017); in brief, sequencing depths of the genomes are 15× or above except that of green anole (7.1×) and corn snake (13×); and the range of Contig N50 is 2.4–437.3 kb. Thus, the quality of genome is high. We used published vertebrate *Tas1r* genes as query sequences and performed TblastN (Altschul et al., 1990) to search for *Tas1r1*, *Tas1r2*, and *Tas1r3* from the above genomes. Diet information was primarily collected from the Animal Diversity Web (ADW, https://animaldiversity.org/, last accessed February 23, 2017), and when the diet of a species wasn’t included in the ADW, we referred to Zhong et al. (2017) and Baeckens, Van Damme & Cooper (2017).

### *Tas1r* gene predictions

To identify the exons in each *Tas1r* gene, we employed a bioinformatic pipeline similar to the one described in Feng & Zhao (2013) and Shi & Zhang (2006). First, previously reported *Tas1r* sequences were used as queries to conduct TblastN to identify the genomic locations of putative *Tas1r* genes in a genome. Second, the genomic scaffolds containing *Tas1r* were downloaded. Third, exons from *Tas1r1* (accession no. KM091451), *Tas1r2* (accession no. NM_152232), and *Tas1r3* (accession no. KM091452) were used as query exons to conduct the BLAST program (Altschul et al., 1990) with the corresponding scaffold. Fourth, through the above steps, some intact *Tas1r* genes could be found, and they were further used as query sequences to repeat the first to third step in other genome whose gene still needed to be identified. To identify the whole coding regions, we extended the blast hit sequences to both 5′ and 3′ directions along the sequences. All exons were assembled and compared with their query sequences by using ClustalX 1.81 (Thompson et al., 1997), and indels (insertions/deletions) which resulted in premature stop codon were recorded from the alignments. Newly identified *Tas1r* genes were classified as intact, partial, or defective according to the following criteria: First, sequences with no frame-shift mutations were further checked by TMHMM Server v.2.0 (http://www.cbs.dtu.dk/services/TMHMM/, an online server which can predict transmembrane helices in proteins) (Sonnhammer, Heijne & Krogh, 1998) to examine whether the protein transmembrane domains exist or not. If all seven transmembrane domains were observed, the gene was considered intact; if not, it was considered partial. Second, sequences with no frame-shift mutation but which included unknown regions (indicated by “N”) were considered partial. Third, sequences containing frame-shift mutations which result in premature stop codon were defined as defective. At last, sequences would be considered absent if no or too short blast hits (shorter than 100 base pairs) were found and the two neighboring genes adjacent to each *Tas1r* were still could be identified.

### Gene syntenic analysis

When we failed to find the *Tas1r* genes, we tried to identify neighboring genes. If the neighboring genes could be found, we viewed the *Tas1r* genes as absence (Shi & Zhang, 2006; Zhao et al., 2010; Feng & Zhao, 2013). In mouse and most species surveyed, the neighboring genes of *Tas1r1* are *Nol9* and *Zbtb48*; neighboring genes of *Tas1r2* are *Aldh4a1* and *Pax7*, and in *Tas1r3*, they are *Dvl1* and *Cptp*. The sequences of which accession numbers are NM_001159599, NM_133879, NM_011039, NM_175438, NM_010091, and NM_024472 were used as query sequences to identify the neighboring genes of each *Tas1r*.

### Phylogenetic tree reconstruction

The phylogenetic tree was reconstructed by using TimeTree (http://www.timetree.org/), a web-based database which collects literature on divergent time estimates among species and is easy for researchers to learn about the TimeTree of life (Hedges, Dudley & Kumar, 2006). In brief, dataset of species name was put into TimeTree and searched, and the phylogenetic tree was produced. When a species wasn’t included in the tree, we supplemented it by referring to Zhong et al. (2017) and Pyron, Burbrink & Wiens (2013).

## Results

**Figure 1:**
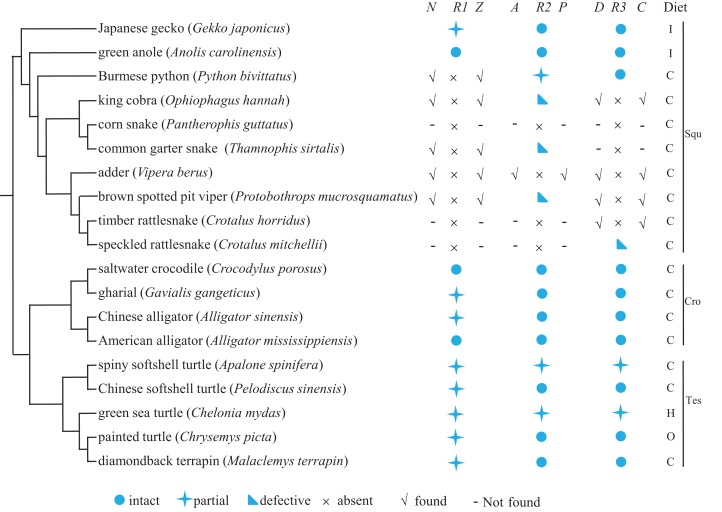
The umami/sweet taste receptor gene functionality of 19 reptiles and their dietary preferences. The distribution of flanking genes are also shown when *Tas1r* gene is absent. The species phylogeny was drawn from the TimeTree (http://www.timetree.org/) and referred to Zhong et al. (2017) and Pyron, Burbrink & Wiens (2013). *N*, *Z*, *A*, *P*, *D*, *C* denotes the neighboring gene *Nol9*, *Zbtb48*, *Aldh4a1*, *Pax7*, *Dvl1*, and *Cptp*, respectively, and *R1*, *R2*, *R3* indicates *Tas1r1*, *Tas1r2*, *Tas1r3*. Squ, Cro, Tes denotes squamata, crocodylia and testudines, respectively.

We explored the evolution of *Tas1r* genes in reptiles by searching *Tas1r1*, *Tas1r2*, and *Tas1r3* in 19 reptiles (comprising ten squamates, five testudines, and four crocodiles) for which the genomic information is currently available. Meanwhile, the functionality of newly obtained sequences was predicted, and feeding preference of each species was searched. Results are shown in Fig. 1. As a whole, our results showed that, 11 *Tas1r1*, 12 *Tas1r2*, and 12 *Tas1r3* were identified to be intact or partial (see Raw Data); 3 *Tas1r2* and 1 *Tas1r3* were pseudogenes, and 5 *Tas1r1*, 1 *Tas1r2*, and 4 *Tas1r3* were absent with their flanking genes presence (Fig. 1 and Table S1). Additionally, 3 *Tas1r1*, 3 *Tas1r2*, 2 *Tas1r3*, and their respective neighboring genes were absent. The functionality of umami/sweet receptor genes varied among the different reptile lineages. Specifically, in the squamata, *Tas1r1* is intact in green anole and partial in Japanese gecko, and *Tas1r1* of all eight snakes appears to be missing. The neighboring gene of *Tas1r1* (*Nol9* and *Zbtb48*) can be identified in five out of the eight snakes, that is, Burmese python, king cobra, common garter snake, adder, and brown spotted pit viper, which are from different snake lineages. Thus we speculated that perhaps all snake *Tas1r1* genes are lost. *Tas1r2* is partial in Burmese python and pseudogenized in king cobra, common garter snake and brown spotted pit viper (Fig. 1; Table 1; and Fig. S1) while it is absent in corn snake, adder, timber rattlesnake and speckled rattlesnake. The *Tas1r2*’s neighboring gene *Aldh4a1* and *Pax7* can be identified in adder, however, they are absent in corn snake, timber rattlesnake and speckled rattlesnake. *Tas1r2* is intact in green anole and Japanese gecko. *Tas1r3* is intact in Burmese python and pseudogenized in speckled rattlesnake but absent in other six snakes, with flanking genes *Dvl1* and *Cptp* remaining presence in king cobra, adder, brown spotted pit viper and timber rattlesnake (Fig. 1 and Table S1) but absence in corn snake and common garter snake, whereas it is intact in green anole and Japanese gecko. In crocodylia species, *Tas1r1* is partial in gharial and Chinese alligator while it is intact in saltwater crocodile and American alligator. Both *Tas1r2* and *Tas1r3* are intact in these four species. As for the testudines, *Tas1r1* is partial in all five species. Both *Tas1r2* and *Tas1r3* are intact in Chinese softshell turtle, painted turtle, and diamondback terrapin but partial in spiny softshell turtle and green sea turtle. Among the results mentioned above, it is worth pointing out that, within the squamata lineage, the umami/sweet taste receptor gene evolution is different in lizards and snakes. The lizards maintain umami/sweet taste perception (except that the *Tas1r1* is partial in Japanese gecko), however, all the snakes possibly lose the umami/sweet taste perception except for the sweet taste to Burmese python, indicative of weak umami/sweet taste function in snakes.

**Table 1:** Indels (insertions/deletions) and premature stop codon numbers of defective *Tas1r* genes in reptiles used in this study.

Species	Exon1		Exon2		Exon3		Exon4		Exon5		Exon6		No. of premature Stop codon
	Insertion	Deletion	Insertion	Deletion	Insertion	Deletion	Insertion	Deletion	Insertion	Deletion	Insertion	Deletion	
*Tas1r2*													
King cobra	–	–	–	–	1 bp	0 bp	–	–	–	–	1, 1, 1 bp	1, 2, 2, 8 bp	2 at exon3; 3 at exon6
Common garter snake	–	–	0 bp	7 bp	–	–	–	–	–	–	–	–	1 at exon2
Brown spotted pit viper	–	–	0 bp	0 bp	–	–	–	–	–	–	1 bp	1, 1 bp	1 at exon6
*Tas1r3*													
Speckled rattlesnake	1 bp	2, 1 bp	–	–	–	–	–	–	–	–	–	–	1 at exon1

**Note:**

“–” Indicates no available information.

When relating the dietary preference to the functionality of *Tas1r* genes (Fig. 1), we found that no correlation existed between feeding ecology and *Tas1r* evolution in reptiles. In details, among the squamate lineage, although all the species studied here are carnivorous or insectivous, the *Tas1r* evolution varies between snakes and lizards. That is, the majority of snake *Tas1r* genes are absent or pseudogenized, in contrast, *Tas1r* genes are present in lizards. In the crocodilians, all the four species are carnivorous, but *Tas1r* genes are intact in both saltwater crocodile and American alligator while *Tas1r1* is partial in gharial and Chinese alligator, and *Tas1r2* and *Tas1r3* are intact in these two species. In testudines, *Tas1r1* of all the five species are partial due to the failure of identifying exon1, and it is likely because the exon1 length is very short (152 bp) and the sequencing quality in these regions is poor. Additionally, gene loss or pseudogenization can occur in all the snake lineages studied here. For instance, *Tas1r1* absence could possibly occur in all the snakes, and *Tas1r2* pseudogenization can happen in viperidae, colubridae, and elapidae which are different lineages of snakes. Taken all together, the feeding preference has no relationship with the evolution of *Tas1r* genes in the reptiles studied here.

## Discussion

We have explored the evolution of *Tas1r* genes in the major groups of reptiles, and analyzed the correlation between *Tas1r* genes functionality and feeding ecology. The evolution of *Tas1r* genes is different among the lineages of reptiles. Among the reptiles, squamate lineages, especially the snake lineage, tends to lose umami/sweet taste perception more easily than crocodylia and testudines lineages. The results revealed that the majority of snakes possibly have lost their umami/sweet taste perception, and gene loss may happen in any snake lineage. When considering the relationship between evolution of *Tas1r* genes and the dietary preference, most of the reptile species are carnivorous, but their *Tas1r* genes can be absent, pseudogenized, partial, or intact, suggesting that no correlation exists between *Tas1r* functionality and feeding ecology, and dietary preferences isn’t a driving force of *Tas1r* evolution. The result agrees with Zhao & Zhang (2012), which suggests the taste receptor evolution and feeding preferences are not matched. The conclusion in Zhao & Zhang (2012) mainly focuses on mammals and birds, and conclusion of this study is based on reptiles.

Additionally, although the sequencing quality of genome is good, it is inevitable that some regions can’t be successfully sequenced due to the complexity of sequence. Thus, when neither *Tas1r* genes nor flanking genes can be identified, we speculate that the genes may be lost but this cannot be conclusively determined.

Combining our result of *Tas1r* evolution and the information on *Tas2r* evolution from Zhong et al. (2017), it is suggested that the *Tas1r* genes functionality is consistent with the variation of *Tas2r* number. In crocodylia and testudines, all the species have intact or partial *Tas1r* genes. Their total *Tas2r* gene number varies from 5 to 18, and intact gene number varies from 2 to 11. In squamata, *Tas1r* genes are intact or partial in lizards, accordingly, the total and intact *Tas2r* gene number ranges are 50–70 and 36–50, respectively. However, the most striking is the snake lineage: most snakes appear to have lost Tas1r function; correspondingly, their *Tas2r* gene numbers are contracted dramatically, with total number from 2 to 3, and intact gene number only 1–2. In contrast, the number of functional *Tas2r* genes has a significant positive correlation with feeding preference, while *Tas1r* functionality doesn’t correlate with feeding ecology in reptiles.

It has been proposed that dietary and foraging pattern of swallowing food whole without chewing may account for the contraction of *Tas2r* gene numbers in snakes (Zhong et al., 2017). However, why do the snakes tend to lose umami/sweet taste perception more easily? Considering that all the snakes appeared to have lost their umami taste perception, the majority of snakes seemed to have lost sweet taste perception and the bitter taste receptor genes are also reduced dramatically in the snakes (Zhong et al., 2017), we put forward three possible explanations. First, snakes are vomeronasal specialists (Schwenk, 1993, 1995) and both the olfactory and vomeronasal receptors genes are expanded in snakes. Thus, other sensory systems may compensate for weak taste perception during foraging (Castoe et al., 2013; Zhong et al., 2017). Second, anatomical evidence supports the absence or pseudogenization of *Tas1r* genes in the snakes. Previous research (Schwenk, 1993) found that taste buds of serpents are absent, which leads to the absence or reduction of taste receptor genes in snakes as most taste receptors are attached to taste cells of the buds; in contrast, the taste buds of Iguanidae (green anole) and Gekkonidae (Japanese gecko) are present (Schwenk, 1993), limiting impact on the taste receptor genes. Third, it is suggested that swallowing food whole without chewing may account for the taste loss in some marine mammals (Jiang et al., 2012; Feng et al., 2014), and snakes have the same forage pattern of swallowing food whole. The pattern of taste loss along with a similar forage pattern in marine mammals supports the potential of similar mechanisms operating in *Tas1r* evolution in reptiles.

During the data mining process, we found that the gene annotations of reptiles are sometimes incorrect in Ensembl (http://asia.ensembl.org/index.html). For instance, *Tas1r2* of the Chinese softshell turtle is absent in Ensembl, but our results show that it is intact. Moreover, incomplete genome sequencing exists in some species. For example, multiple “N” exists in the *Tas1r3* of spiny softshell turtle and green sea turtle.

In sum, the relationship between *Tas1r* functionality and diet is complicated. *Tas1r* functionality is divergent among reptile lineages in that the majority of *Tas1r* genes in snakes appears to be absent or pseudogenized while in crocodylia and testudines they are partial or intact. Furthermore, according to the previous study, sequencing errors could occur in the publicly available genome database (Feng & Zhao, 2013), and draft genome sequences are not sufficient to conclude whether a gene is intact or defective, thus the functionality of *Tas1r* genes in reptiles should be checked by re-sequencing in the future. Lastly, to make clear the driving forces for *Tas1r* evolution in reptiles, future work on more accurate and complete functional characterizations of taste receptor genes is needed.

## Conclusion

Our study mainly explored the evolution of *Tas1r* and the correlation between *Tas1r* evolution and the feeding preferences in 19 reptile species. The results suggest that the *Tas1r* evolution is different among reptile lineages, and that there is no correlation between *Tas1r* evolution and feeding preferences. In particular, it is likely that many snakes completely lost their *Tas1r* genes or the umami/sweet taste function. We inferred that the well-developed vomeronasal system, the absence of taste buds and the feeding manner of swallowing food whole may account for the loss of the umami/sweet taste in the snakes. Finally, gene functionality inferred only from the genome or the public database is not enough, and more accurate conclusions should be draw from re-sequencing or even functional experiments.

## Supplemental Information

10.7717/peerj.5570/supp-1 Supplemental Information 1 Fig. S1. Nucleotide sequence containing the ORF-disrupting mutation. The query sequence is shown above and the number in parentheses indicates the start nucleotide. The codon which includes frame-shift mutation is marked by a red box.

10.7717/peerj.5570/supp-2 Supplemental Information 2 Table S1. Syntenic location for the flanking gene of missing *Tas1r* genes. *Nol9* and *Zbtb48*, *Aldh4a1* and *Pax7*, *Dvl1* and *Cptp* are the flanking genes of *Tas1r1*, *Tas1r2* and* Tas1r3*, respectively. Numbers in the parentheses denote the amino acid length of gene. “-”indicates no *Tas1r* was detected.

10.7717/peerj.5570/supp-3 Supplemental Information 3 Raw data for the *Tas1r* genes identified in this study. *Tas1r1* of spiny softshell turtle is omitted due to the large numbers of “N” containing in it.

## Additional Information and Declarations

### Data Availability

The following information was supplied regarding data availability: The raw data are provided in the Supplemental Files.
