# Supplementary material for: Molecular evolution of umami/sweet taste receptor genes in reptiles
Source: PeerJ. 2018 Aug 24;6:e5570. doi: 10.7717/peerj.5570 (PMC6110252; doi:10.7717/peerj.5570)
Supplement: Supplemental Information 1 — The query sequence is shown above and the number in parentheses indicates the start nucleotide. The codon which includes frame-shift mutation is marked by a red box. [file peerj-06-5570-s001.pdf]

Tas1r2 Exon 2

```
Human               (292)  5'  ATCGTGGATGTGTGCTACATCTCCAACAATGTCCAGCCGGTGCTCTACTTCCTGGCACAC  3'
common garter snake        5'  ATGATGGATATCTGCTTCCTCACCAA-------CACCCCATCCTGCATTTCCTCGCTGAC  3'
```

Tas1r2 Exon 3

```
Human       (910)  5'  TGGGCCATCGACCCGGTCCTGCACAACCTC-ACGGAGCTGCGCCACTTGGGCACCTTCCTG  3'
king cobra         5'  TGGGCTGCTGACCTGTCCATCCACGGCCTCTGCAACATTTCCAGGCTGGGAGACGTATTTG  3'
```

Tas1r2 Exon 6

```
Human       (1840)  5'  GCATACATGGTGGTCCCGGTGTACGTGGGGCCGCCCAAGGTCTCCACCTGCCTCTGCCGCCAGGCCCTCTTTCCCCTCTGCTTCACAATC  3'
king cobra          5'  GCTTCTGCAGTG-TCTTCTCCTATGTTGGAGTCCCAACCGAGCTGAGGTGTCTTTTCTGCCTGGCCAGGTACAGCCTCTGCTTCACCATC  3'

Human               5'  TGCATCTCCTGTATCGCCGTGCGTTCTTTCCAGATCGTCTGCGCCTTCAAGATGGCCAGCCGCTTCCCACGCGCCTACAGCTACTGGGTC  3'
king cobra          5'  TGCCTTGCCTGCGCTGCGATCCGTTCCATCCGGATCCTCTGCGCTTTCAAGATGGTGGCCTGGCTGCCTGCAACCTTCATCTCCTGGTCC  3'

Human               5'  CGCTACCAGGGGCCCTACGTCTCTATGGCATTTATCACGGTACTCAAAA-TGGTCATTGTGGTAATTGGCATGC-TGGCCACGGGCCTCA  3'
king cobra          5'  ACGTCCAAAGGGCAGCGGGTTTTCCTCGCCACCATTTCCGCCATCAAAGGTGGCCACCGTGATACTCAACCTCCATTACCGCTCCCCGAA  3'

Human               5'  GTCCCACCAC-CCGTACTGACCCCGATGACCCC---AAGATCACAATTGTCTCCTGTAACCCCAACTACCGCAACAGCCTGCTGTTCAAC  3'
king cobra          5'  GCCCGTCCAGGCTGCCCTAATG--GGTAACCCGGCCGAGCTTTCCCTGACTTGTCACAAC--CGGCGACCTGTCCGTGGTTGTGCTCAAC  3'

Human               5'  ACCAGCCTGGACCTGCTGCTCTCAGTGGTGGGTTTCAGCTTCGCCTACATGGGCAAAGAGCTGCCCACCAACTACAACGAGGCCAAGTTC  3'
king cobra          5'  CATCTCTTTGACATGGTCCTCTCCTCCCTGGGCTTCTGCTTGGCCTATGCGGGGAAGGTGTTGCCAAAGAGCTACAGTGAAACCAAGTTC  3'

Human               5'  ATCACCCTCAGCATGACCTTCTATTTCACCTCATCCGTC  3'
king cobra          5'  ATCTCCATCA--------TCCTGCTTCAGCTCCTGGGTG  3'
```

Tas1r2 Exon 6

**Human**	(1780)	5’	CCCATAGTTCGCTCGGCTGGGGCCCCATGTGCTTCCTGATG-CTGACACTGCTGCTGGT	3’
**brown spotted pit viper**		5’	CTGGTGGTGAGATCGGCCGGTGGCAGGCTGTGTTTCCTCATGTCCTCCTCCCTTGTCTTC	3’
**Human**		5’	GGCATACATGGTGGTCCCGGTGTACGTGGGGCCGCCCAAGGTCTCCACCTGCCTCTGCCG	3’
**brown spotted pit viper**		5’	GGCTTCTGCAGTG-TCTTCTCCTATGTGGGGTC-CCCACCGAGCTGAGATGTCTCTTCCG	3’
**Human**		5’	CCAGGCCCTCTTTCCCCTCTGCTTCACAATC	3’
**brown spotted pit viper**		5’	CCTGGCCGGCTTCACCGTCTGCACCACCGTC	3’

Tas1r3 Exon 1

**Chicken**	(1)	5’	ATGATCCCCTGGGTGCTGCTGTGTATGAGCTTCGGTTGCGCAGCAGCCCTGAAGCCCAGC	3’
**speckled rattlesnake**		5’	ATGATATTT--GTTAGTGGTCAGGTTTGCTTTCAATGGTGCCGTAGCCCTGCAACACAAC	3’
**Chicken**		5’	TGCCTCTCGGCTCAGTTTAGGAGGCCTGGTGACTACATCATTGGAGGCCTGTTCCCTTTT	3’
**speckled rattlesnake**		5’	TGCTTGTCTTCCCAGTTTAGAAGGCCTGGAGACTACGTCTTGGGTGGGCTGTTCCCATTC	3’
**Chicken**		5’	GGGATGGACACCATCAACCTGAC-AGCACGATCAGAGCCCACCTTAATCGTGTGTGAA	3’
**speckled rattlesnake**		5’	AGAATTGGCACTACCAAGCTGTCCAGCCAGACTCTGCCAGAGCTTCACAAC-TGTGGG	3’
