# Supplementary material for: Molecular evolution of umami/sweet taste receptor genes in reptiles
Source: PeerJ. 2018 Aug 24;6:e5570. doi: 10.7717/peerj.5570 (PMC6110252; doi:10.7717/peerj.5570)
Supplement: Supplemental Information 2 — Nol9 and Zbtb48, Aldh4a1 and Pax7, Dvl1 and Cptp are the flanking genes of Tas1r1, Tas1r2 and Tas1r3, respectively. Numbers in the parentheses denote the amino acid length of gene. “-”indicates no Tas1r was detected. [file peerj-06-5570-s002.docx]

**Table S1 Scaffold and gene location of the flanking genes.**

| Species | Gene | Scaffold | Position Query | Position Subject | Position Query | Position Subject |
| --- | --- | --- | --- | --- | --- | --- |
|  | *Tas1r1* |  | ***Nol9* (714aa)** |  | ***Zbtb48* (681aa)** |  |
| Common Garter Snake | - | LFLD01S003314.1 | 307-670 | 16339-28756 | 1-584 | 37090-43420 |
| Adder | - | KN638042.1 | 151-670 | 161950-167201 | 1-679 | 155198-159597 |
| Brown Spotted Pit Viper | - | LD637054.1 | 143-670 | 303787-308992 | 1-679 | 311636-316170 |
| King Cobra | - | AZIM01003385.1 | 149-670 | 10755-19887 | 3-679 | 22550-30077 |
| Burmese Python | - | KE957154.1 | 151-710 | 27665-37765 | 3-679 | 14391-22351 |
|  |  |  |  |  |  |  |
|  | ***Tas1r2*** |  | ***Aldh4a1* (562aa)** |  | ***Pax7* (503aa)** |  |
| Adder | - | KN616455.1 | 20-562 | 94046-107939 | 260-503 | 4890-56539 |
|  |  |  |  |  |  |  |
|  | ***Tas1r3*** |  | ***Dvl1*(695aa)** |  | ***Cptp* (216aa)** |  |
| Timber Rattlesnake | - | LVCR01025734.1 | 447-695 | 92-4420 | 1-216 | 10784-12869 |
| Adder | - | KN628019.1 | 1-695 | 106433-139608 | 1-216 | 97714-99564 |
| Brown Spotted Pit Viper | - | LD637301.1 | 1-695 | 281024-324221 | 1-216 | 330574-332913 |
| King Cobra | - | AZIM01001950.1 | 1-695 | 105301-170347 | 39-216 | 179508-180035 |
